# Carotid endarterectomy using regional anesthesia: technique and considerations

**DOI:** 10.3389/fsurg.2024.1421624

**Published:** 2024-06-06

**Authors:** Varun Padmanaban, Catherine Caldwell, Indigo Milne, Sprague W. Hazard, Robert E. Harbaugh, Ephraim W. Church

**Affiliations:** ^1^ Department of Neurosurgery, Pennsylvania State University College of Medicine, Penn State Milton S. Hershey Medical Center, Hershey, PA, United States; ^2^ Department of Anesthesia and Perioperative Services, Pennsylvania State University College of Medicine, Penn State Milton S. Hershey Medical Center, Hershey, PA, United States

**Keywords:** carotid disease, carotid endarterectomy, regional anesthesia (RA), surgical technique, awake carotid endarterectomy (CEA)

## Abstract

**Background:**

Carotid endarterectomy (CEA) is one of the most effective operations in minimizing stroke risk in both symptomatic and asymptomatic patients with carotid stenosis in the United States. Awake CEA with regional anesthesia may decrease both perioperative complications and length of hospital stay. Techniques of performing awake CEA is not often described in published literature.

**Objective:**

To describe our experience with CEA using regional anesthesia with a focus on patient selection, anatomic variations, and surgical technique including cervical regional block. We particularly focus on nuances of the awake approach.

**Methods:**

CEA using regional anesthesia is described in detail.

**Results:**

Successful use of regional anesthesia during CEA without complication.

**Conclusion:**

Regional anesthesia for CEA is an advantageous approach for cervical plaque removal in appropriate patients. Thoughtful patient selection, as well as understanding of anatomy and its variants, is required. Potential advantages and disadvantages are discussed.

## Introduction

Stroke is among the leading causes of death and accounts for a large portion of healthcare costs in the United States and globally (1). Roughly 10% of ischemic cerebral infarctions are due to extracranial atherosclerotic cerebrovascular disease (2). Removal of atherosclerotic plaque causing stenosis of internal carotid arteries (ICA) through carotid endarterectomy (CEA) is arguably the most effective operation in minimizing stroke risk in both symptomatic and asymptomatic patients with carotid stenosis, with a number needed to treat of approximately 6 and 19 respectively (3, 4).

CEA may be performed under either regional or general anesthesia. Regional anesthesia allows for at least equivalent results in patients who are unable to tolerate general anesthesia (5–7) and may be associated with shorter hospital length of stay, reduced anesthesia and operative time, decreased complications including pneumonia and cranial nerve injury, and lower costs (8–15). Furthermore, there may be advantages in patients with contralateral carotid occlusion (5). In this technical paper we review the importance of patient selection, anatomical variations, and operative nuances including cervical block for performing CEA under regional anesthesia. Appropriate informed consent was obtained for surgical photos.

### Review of anatomy

A nuanced understanding of the cervical plexus and its variations is essential to performing carotid endarterectomy under regional anesthesia. Sensation to the skin and superficial structures of the anterolateral neck is supplied by the superficial or cutaneous cervical plexus. It has origins from the ventral rami of nerve roots C2–C4. These branches exit at the midpoint along the posterior border of the sternocleidomastoid muscle, superficial to the prevertebral fascia at approximately the level of the thyroid cartilage posterior to the external jugular vein. These roots form four major terminal branches: The lesser occipital (C2), greater auricular (C2-3), transverse cervical (C2-3) and supraclavicular nerves (C3-4). The deep or muscular cervical plexus innervates the deeper structures of the neck, including muscles of anterior neck and diaphragm via the ansa cervicalis, phrenic and segmental branches. The carotid artery itself, including the carotid bulb, is innervated by the carotid branch of the glossopharyngeal nerve, eponymously named Hering's nerve.

One may encounter many anatomic variations during surgery, and an appropriate preoperative assessment including various imaging modalities should be used to prepare for the individual patient. A poor initial response to cervical block may indicate variations within sensory branches of the cervical plexus. These should be kept in mind when performing the block.

## Operative technique

### Preoperative workup

Patient selection in awake CEA is similar to CEA with general anesthesia including symptomatic carotid artery disease with 50%–99% stenosis (by stenosis criteria determined by the North American Symptomatic Endarterectomy Trial) (4). Asymptomatic patients with severe stenosis (70%–99% stenosis) may be selected on a case-by-case basis after strong consideration for medical management (16). Those who have suffered a large, debilitating stroke may benefit from delayed CEA. In patients who suffered a transient ischemic attack or mild to moderate stroke, urgency may be warranted given the increased risk of stroke in the first weeks after the initial event. Preoperative evaluation should include a careful history and physical examination, as well as preoperative imaging. Patients with language or cognitive deficits may not tolerate regional anesthesia. History of previous neck surgery may warrant evaluation of the recurrent laryngeal nerve.

Discussing peri- and postoperative expectations regarding regional anesthesia is of paramount importance. The procedure must be done expeditiously but may still cause significant anxiety and physical discomfort. While the senior authors performs most CEAs awake with cervical block, some patients are too anxious to benefit from this approach. We collaborate with our patients to decide together on regional vs. general anesthesia, and we find most patients strongly prefer the awake approach citing a fear of general anesthesia and an interest in participating in the surgery to ensure the best possible outcome. Table 1 reviews relative contraindications to performing awake vs. asleep CEA.

**Table 1:** Relative contraindications to performing asleep versus awake CEA.

Awake CEA	Asleep CEA
• Morbid obesity • Obstructive sleep apnea • Severe back/neck pain • Claustrophobic patients • Surgeon discomfort • Neurological deficit precluding intra-operative exam • Non-English speaker	• High anesthesia risk • Hemodynamic instability

### Preoperative planning

Under regional anesthesia, maximizing comfort for the patient while in the operating room is key to the success of the operation. We generally refrain from using central and arterial lines, urinary catheter, and sequential compression devices unless their use is imperative to the performance of a safe procedure. Minimizing anxiety of the patient is requisite for the success of CEA under local anesthesia. We encourage conversing with the patient throughout the operation, and music may be played to the patient's preference. Keeping the atmosphere of the operative room calm and light ensures favorable operative conditions for the surgeon and an improved patient experience. The attending surgeon may need to take time at the beginning of the case and even pause throughout the case to create and maintain this atmosphere.

### Operation

**Figure 1:**
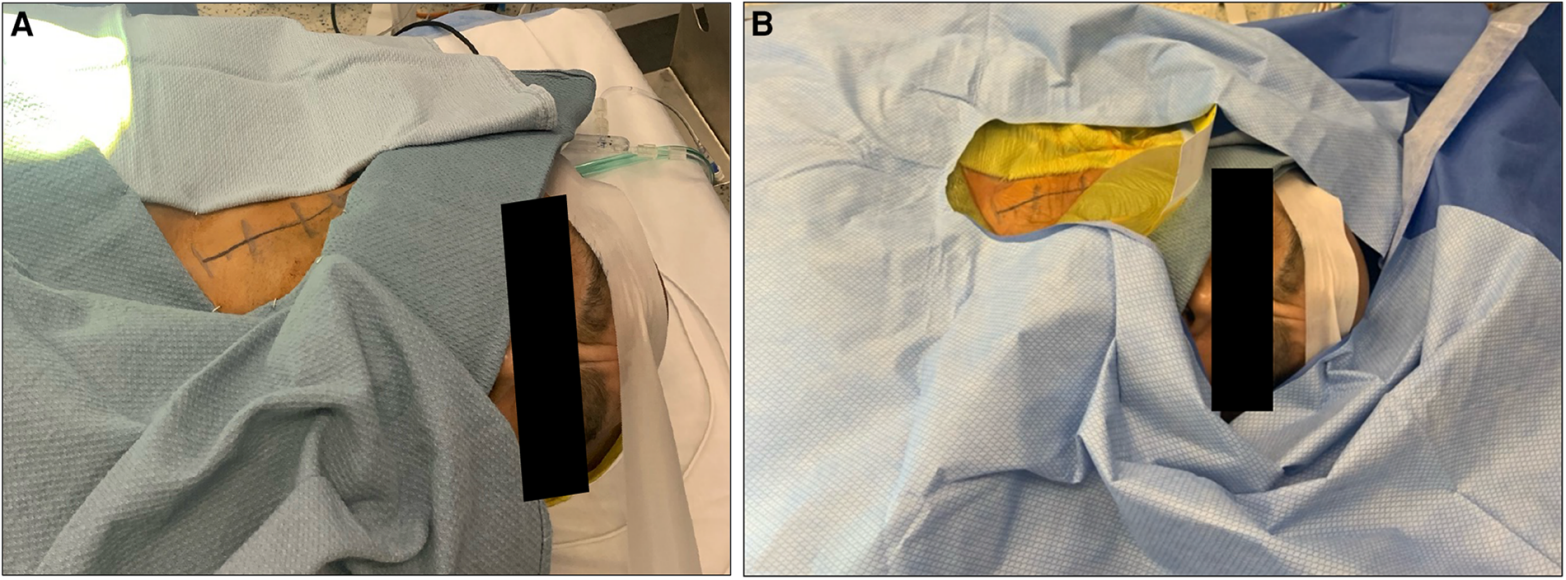
Appropriate positioning and draping for a patient undergoing awake carotid endarterectomy. Note positioning of surgical towels (**A**) and drapes (**B**).

**Figure 2:**
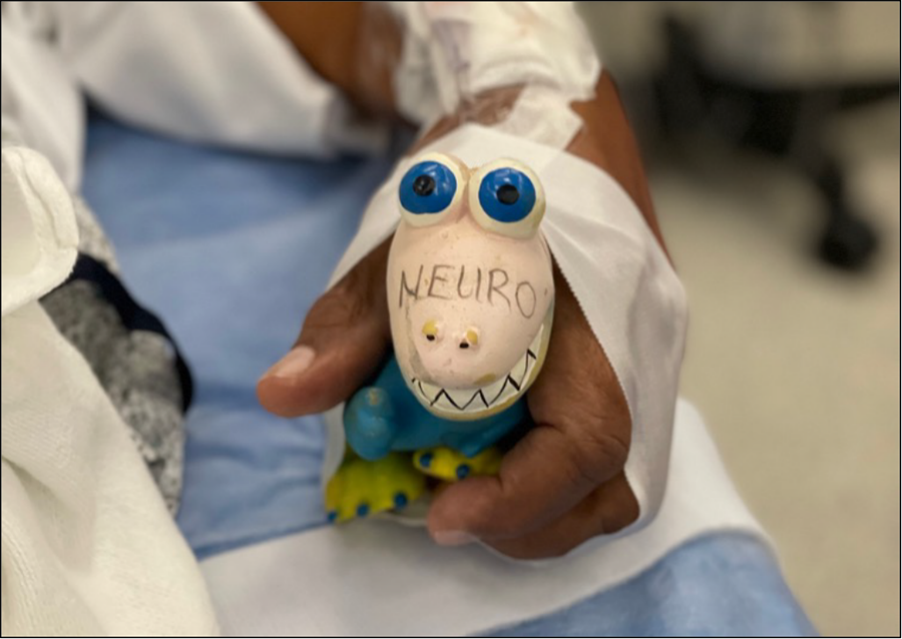
Sample of auditory device taped to patient's hand contralateral to affected carotid artery.

The patient is positioned supine with the head facing away from the side of the affected vessel. A large, folded pillow is placed under the patient's neck to achieve adequate extension and elevation without compromising patient comfort. An elevated gel donut supports the patient's head. Draping between surgeon and anesthesiologist is kept at waist level and drapes are kept away from the patient's face in order to maximize communication and minimize anxiety (Figures 1A,B). An insufflated oblong device with an audible pressure release valve (e.g., squeaky toy) is taped to the patients hand contralateral to the affected carotid. This is used for neurological testing during the operation (Figure 2). Blood pressure is generally maintained at the patient's baseline, and may be elevated 10% during ICA clamping on a case-by-case basis. A forced air patient warming system may be utilized to help regulate the patient's temperature during the case and promote normothermia.

**Figure 3:**
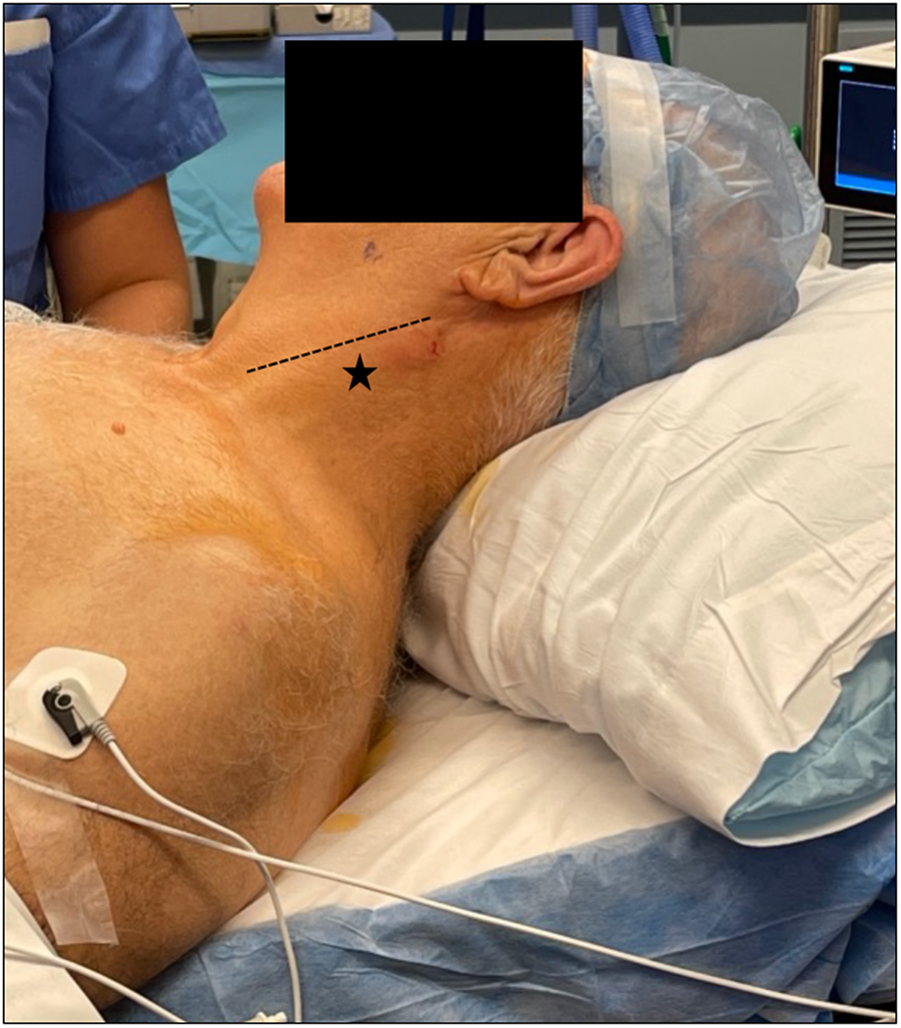
Appropriate starting point of cervical block (star) approximately one third to two thirds along the posterior border of the sternocleidomastoid (line).

The surgeon or the anesthesiologist may perform the cervical field block. Regional anesthesia is achieved with a superficial cervical block utilizing 0.5% lidocaine. Maximum dosage (approximately 4.5 mg/kg) should be noted to avoid potential systemic toxicity. To obtain adequate blockage, a generous volume is injected along one third to two thirds of the posterior aspect of the sternocleidomastoid muscle fanning superiorly and inferiorly (star, Figure 3). Ultrasound may be utilized to visualize the cervical plexus, although the plexus is not always easily visible, and ultrasound is not necessary in most cases. In our experience, maximizing the overall volume of lidocaine is more important than concentration, therefore we dilute our 0.5% lidocaine 1:1 in normal saline (to form 0.25% lidocaine). Minimal sedation should be involved to prevent patient confusion and ensure accurate neurological monitoring. However, low basal drips of dexmedetomidine and remifentanil may be helpful for highly anxious patients. It should be noted that dexmedetomidine may cause blood pressure and heart rate changes, while remifentanil may cause respiratory depression.

A transverse incision may be beneficial for cosmetic outcome and is placed 2 finger breadths below the mandible from 1 cm off midline to 1 cm behind the angle of the mandible. The incision is tucked in a dominant skin crease if possible. A vertical incision along the mesial border of the sternocleidomastoid muscle may also be used, particularly in the case of high carotid bifurcation or when shunting is anticipated. In the senior authors' experience, there are very few bifurcations that cannot be readily reached with a vertical incision that curves slightly posterior at its superior extent. Local anesthetic should be injected along the incision line, particularly at the cephalad aspect where the cervical block may not be as effective.

The opening is performed in the standard fashion along the anterior border of the sternocleidomastoid muscle, mesial to the jugular vein, to the carotid sheath. The vagus nerve runs between the jugular vein and the common carotid artery (CCA) and is carefully protected. Working cephalad, the common facial vein may be ligated and cut. At this point the medial retractor blade must stay superficial to prevent injury to the recurrent laryngeal nerve. The hypoglossal nerve is commonly found beneath or adjacent to the posterior belly of the diagastric muscle and must be carefully protected. While the diagastric may be retracted superiorly with a hook, the nerve must be treated with care. There is commonly a contribution from the hypoglossal to the vagus. Cutting this allows further exposure of the ICA to the skull base if necessary.

We typically maintain patients on dual antiplatelet medications and also administer heparin at 100 U/kg prior to dissection of the ICA. Patients often complain of a deep visceral pain during this portion of the dissection which can be treated with a small dose of lidocaine injected into the carotid sheath at the bulb. A very small IV dose of a short acting opioid can also be utilized. After optimizing blood pressure, clamps and clips are placed in the standard fashion beginning with the ICA and ending with the external carotid artery (ECA). Throughout the clamp time the patient should be tested for motor function by squeezing the toy and, when operating on the dominant side, speech function through conversation.

It is essential to be prepared for a smooth transition to shunt should there be changes in the neurological exam. In the awake setting, this can lead to stress for the patient and the team, and a well-planned and rehearsed shunt placement will keep all at ease. Two loops of umbilical tape are placed around the CCA and a single loop is placed around the ICA, allowing for the Rummel clamp should a shunt be required. A long silk tie should be secured around the shunt to keep it from moving within the vessel out of the reach. The senior authors prefers to use an Argyle shunt and place the shunt in the distal ICA first. After the Rummel is advanced, the shunt can be backbled before placing it in the CCA. This approach minimizes blood loss, keeps the field clear, and keeps the team and the patient calm. To remove the shunt, two snaps are placed through the remaining arteriotomy, and the shunt is cut between the clamps. The two ends are removed sequentially as the vessels are again clamped for the final closure.

The endarterectomy is performed in the standard fashion. We cut the plaque sharply in the CCA, extricate it from the ECA and feather the endarterectomy in the ICA. If the plaque does not feather nicely in the ICA, it can be tacked to the endothelium with double arm prolene stitches. After back bleeding the ICA, the arteriotomy is closed in the standard fashion. We use a patch on an as needed basis.

It is common to rush the closure in an awake patient, and this temptation must be resisted. To obtain hemostasis, half the heparin dose is reversed with protamine at 10 min post clamping. Meticulous coagulation and application of hemostatic materials is paramount. The sternocleidomastoid muscle and platysma are re-approximated to close open spaces where hematoma may form.

## Discussion

### Key results

Carotid endarterectomy with regional anesthesia has continued as a highly effective approach to the CEA operation (8–11). A prospective randomized study as well as subgroup analysis of the Stenting vs. Endarterectomy for Treatment of Carotid Artery Stenosis trial comparing the two techniques reported that the combined rates of cerebral infarction, myocardial infarction, and perioperative death were similar for local/regional anesthesia vs. general anesthesia, while local/regional anesthesia resulted in better outcomes for patients who had occlusion of the contralateral internal carotid artery (5, 6). A recent Cochrane meta-analysis has also shown equivalence between the two methods (17). Multiple, smaller studies have shown that regional anesthesia is associated with decreased rates of complications, shorter hospital stays, and lower costs compared to general anesthesia (7–15).

Without neurological monitoring of an awake patient, surgeons must employ either routine use of shunts for all patients undergoing CEA or neurological monitoring via electroencephalogram, cerebral oximetry, transcranial Doppler scanning, and determinations of ICA back pressure (18). Routine use of shunt placement may lead to a higher rate of postoperative stroke/transient ischemic attack (19, 20). Experience with regional anesthesia can help to extend the population that can undergo CEA due to chronic disease that might make general anesthesia risky, including advanced inoperable coronary artery disease, and chronic obstructive pulmonary disease (11).

A common fear in both patients and their surgeons considering regional anesthesia for CEA is agitation during surgery. In our experience, limiting intravenous anesthetic to keep the patient truly awake and ensuring patient comfort is essential. Pausing, stopping and reassessing if the patient is uncomfortable is integral, especially prior to the critical components of the surgery. In truly critical situations, an oral airway and intravenous sedation with dexmedetomidine or remifentanil maybe utilized. An expeditious operation is also integral to ensuring the patient can tolerate the entire procedure.

A few patients may not tolerate awake surgery well due to personality features and personal preference. A candid preoperative discussion with realistic expectations is essential. While we strongly prefer the awake approach and recommend this to most patients, some patients are not well suited for regional anesthesia and may do better with general anesthesia with neurophysiological monitoring.

### Conclusion

Regional anesthesia for CEA is an advantageous approach for cervical plaque removal in appropriate patients due to its association with fewer complications, shorter hospital stays, and lower costs. A strong patient-physician team, in addition to thoughtful considerations to maximize patient comfort, are critical to allow for minimized anxiety throughout the operation. Good patient selection, mastery of normal and abnormal anatomy, thorough cervical block, and comfort with complication management and technical nuances as detailed above will yield excellent outcomes in awake CEA.

## Data Availability

The original contributions presented in the study are included in the article/Supplementary Material, further inquiries can be directed to the corresponding author.
